# Stimulation with Peptidoglycan induces interleukin 6 and TLR2 expression and a concomitant downregulation of expression of adiponectin receptors 1 and 2 in 3T3-L1 adipocytes

**DOI:** 10.1186/1476-9255-6-8

**Published:** 2009-04-06

**Authors:** Kolapo M Ajuwon, William Banz, Todd A Winters

**Affiliations:** 1 Department of Animal Sciences, Purdue University, West Lafayette, IN 47907, USA; 2 Department of Animal Science, Food and Nutrition, Southern Illinois University, Carbondale, IL 62901, USA

## Abstract

**Background:**

Inflammation is a major component of obesity and diabetes, and toll-like receptors (TLRs) play critical roles in the regulation of inflammation and response to pathogen associated molecular patterns (PAMPs) and fatty acids in. Although immune cells such as macrophages are primarily responsible for recognition and clearance of pathogens, adipocytes are also closely involved in the regulation of innate immunity and inflammation. Whereas it has been demonstrated that adipocytes respond to TLR4 stimulation with lipopolysacccharide, very little is known about their response to the TLR2 agonist, peptidoglycan.

**Methods:**

We investigated the response to peptidoglycan from *Staphylococcus aureus *in differentiated 3T3-L1 adipocytes. Real-time PCR analysis was used to quantify the expression of interleukin 6 (IL6), adiponectin receptors (adipoR1 and adipoR2), toll-like receptor 2 (TLR2) and 4 (TLR2 4). Media level of IL6 was determined with ELISA.

**Results:**

Adipocyte stimulation peptidoglycan induces IL6 expression (P < 0.01). Both siRNA mediated suppression of TLR2 and immunoneutralization of TLR2 with a TLR2 specific antibody inhibited response to peptidoglycan (P < 0.05). We also examined the regulation of TLR2 and TLR4 mRNA in peptidoglycan treated cells. Both peptidoglycan and lipopolysaccharide (LPS) robustly induce TLR2 mRNA expression, whereas TLR4 mRNA is weakly induced by LPS only (P < 0.05). Additionally, peptidoglycan downregulates the mRNA expression of adiponectin receptors, adipoR1 and adipoR2 (P < 0.05).

**Conclusion:**

Obesity and type 2 diabetes are associated with increased expression of TLR2, this receptor could play a significant but previously unrecognized role in the establishment of chronic inflammation in adipose tissue in obesity.

## Background

Adipocytes are integral components of the overall body innate immune response. This response is mediated mostly by the highly conserved pattern recognition receptors such as toll-like receptors (TLRs) and scavenger receptors [1-3]. These receptors and the signaling cascades that they initiate are also involved in the perpetuation of chronic inflammatory milieu that characterizes obesity and high fat feeding. Thus, they represent attractive targets to prevent obesity-induced metabolic impairments, notably insulin resistance and cardiovascular complications [4]. Toll-like receptor 4 remains the most studied TLR in adipocytes. Our work and that of others have demonstrated that adipocytes respond to inflammatory stimuli initiated by LPS, the TLR4 ligand [1,5]. Additionally, we have shown that palmitate, a saturated fatty acid, induces inflammation in adipocytes, and others have shown that fatty acid-induced inflammation in adipocytes is partly mediated by TLR4 [6,7]. Toll like receptor 2 is another member of the TLR family that is constitutively expressed in adipocytes and is rapidly induced by LPS and tumor necrosis factor (TNF) α [5]. However, adipocyte response to fungal zymosan, a recognized ligand for TLR2, has produced mixed results [5,7], and there is no information on the response of adipocyte to peptidoglycan and possible impact on inflammatory cytokine production. Nevertheless, the pattern of expression of TLR2 suggests that this receptor may be an important component of the inflammatory process in obesity. First, TLR2 expression is significantly increased in adipose tissue of type 2 diabetic and obese patients and its expression is upregulated by resistin, an hormone that induces insulin resistance, suggesting TLR2 may be intricately involved in the regulation of inflammation-induced insulin resistance than hitherto recognized [8,9]. Indeed, a recent report indicates that obesity induces a subset of adipocytes to express both TLR2 and TNFα and exposure of adipocytes to zymosan triggers expression of TNFα [10]. Although mixed results have been observed regarding the response of adipocytes to fungal zymosan, current evidence supports a significant role for this receptor in regulating adipose inflammation. Toll-like receptor 2 is the most promiscuous of all the TLRs and is able to recognize multiple ligands such as fatty acids, fungal zymosan and gram positive bacteria components (peptidoglycan and teichoic acid), lipoarabinomanan, bacterial lipopeptides, some LPS variants from gram-negative bacteria, yeast, spirochetes and fungi [11,12]. In addition, this receptor is able to form heterodimers with other TLRs [13]. Although the identities of its ligands in vivo have not been clarified, we explored the possibility that mature adipocytes respond directly *in vitro *to a gram positive bacteria component.

Adiponectin is a protein that plays a critical role in the regulation of glucose and lipid metabolism by increasing glucose uptake in muscle [14], suppressing gluconeogenesis in the liver [15] increasing fatty acid oxidation in the liver and muscle [14,15]. Our earlier work in 3T3-L1 adipocytes [16] and porcine macrophages [17] and that of others in aortic endothelial cell model [18] also provide clear evidence that adiponectin exerts anti-inflammatory roles in multiple cell types partly by inhibition of nuclear factor kappa B (NFκB). Adiponectin exerts its metabolic effects via two isoforms of its receptor (adipoR1 and adipoR2) [19]. The regulation of adiponectin bioactivity is determined at multiples levels including its oligomerization state [20], and the expression level of its receptors [21]. Obesity and insulin resistance are associated with a lower level of circulating adiponectin and reduced concentration of the high molecular weight species [20]. Obesity also causes reduced expression of adiponectin receptors in adipose tissue [22,23]. Thus obesity also causes a state of adiponectin resistance. However, the mechanisms that lead to downregulation of adiponectin receptors in adipose tissue in obesity have not been clarified. Therefore, because obesity is a state of chronic inflammation that is associated with increased expression of TLR2 and TLR4, we also tested the hypothesis that activation of TLR2 and TLR4 in adipocytes represents a mechanistic link between inflammation and downregulation of adiponectin receptors. Because fatty acids are directly implicated in the induction of inflammation in adipocytes via TLR4 activation [7], and circulating fatty acid concentrations are elevated in obesity, we further explored the possibility that fatty acids exert a direct role in the regulation of TLR2 and TLR4 expression, hence indirectly influencing the inflammatory response in adipocytes.

We provide evidence herein that differentiated adipocytes are equipped with the capability to respond directly to innate immune challenge by gram positive bacteria. This causes induction of IL6 production, an upregulation of TLR2 and a downregulation of adiponectin receptors 1 and 2. This additional information represents an extension of our knowledge of the immunological capabilities of the adipocyte and a potential interaction with adiponectin action. A deeper understanding of the mechanisms that regulate TLR2 signaling in adipocytes may contribute to unraveling the causes of obesity-induced inflammation and insulin resistance.

## Methods

### Culture of 3T3-L1 Adipocytes

Cells were obtained from ATCC (Manassas, VA) and cultured according to standard conditions. Cells were propagated in high glucose Dulbecco's Modified Eagles Medium (DMEM) medium (Hyclone, Logan, UT) containing 10% fetal bovine serum under 5% CO2 and in the presence of 0.5% penicillin-streptomycin mixture (Invitrogen, Carlsbad, CA). Two days postconfluence (day 0), cells were induced to differentiate with a medium containing 10% fetal bovine serum, 1.7 μM insulin, 1 μM dexamethasone, and 0.5 mM IBMX for 48 hour. Afterwards fresh media containing only insulin and 10% fetal bovine serum was added for another 48 hours. Subsequent media changes were done every 48 hours with DMEM containing only 10% FBS. Cells are typically used for experiments between day 10–12 post differentiation. Before being used for experiments, cells were rinsed twice with serum-free low glucose (5 mM) DMEM containing 0.1% fatty acid free BSA (treatment media) and kept in this media for 16–24 hours.

### Experimental treatment with LPS and Peptidoglycan

Fully differentiated adipocytes in serum free media were treated with 100 ng/ml lipopolysaccharide (LPS) from E. coli and 10 μg/ml peptidoglycan (PEP) from *Staphylococcus aureus *(Sigma, St Louis, MO) for the indicated time periods. In experiments with polymyxin B, cells were pretreated with 100 μg/ml polymyxin (Sigma) for 30 minutes and then treated with both LPS and peptidoglycan. We immunoneutralized both TLR4 and TLR2 by pretreating cells with 5 μg/ml of azide free functionally active anti-TLR4 and anti-TLR2 antibodies (eBiosciences, San Diego, CA) for 1 hour before LPS and peptidoglycan treatments for additional 6 hours.

### Real-time quantitative RT-PCR

Total RNA from treated cells was extracted with Tri Reagent (Sigma) according to the manufacturer's protocol. The mRNAs were treated with Turbo Free DNA (Ambion, Austin, TX) and reverse transcribed into cDNA using Improm II reverse transcriptase (Promega, Madison, WI). Real-time PCR was performed using iCycler iQ real-time PCR detection system (Bio-Rad) with the Faststart SYBR mix (Roche, Indianapolis, IN). Primers for IL6 were: forward primer, 5'-AACGATGATGCACTTGCAGA-3' and reverse, 5'-GAGCATTGG AAATTGGGGTA-3'. For TLR2, forward primer, 5'-TGCTTTCCTGCTGGAGATTT-3' and reverse primer, 5'-TGTAACGCAACAGCTTCAGG-3'; TLR4, forward primer, 5'-TTCAAGACCAAGCCTTTCAG-3' and reverse primer, 5'-CATAGTCCT TCCATGATAGA-3'. Primers for β-actin were forward, 5'-ATGGGTCAGAAGGAC TCCTACG-3' and reverse, 5'-AGTGGTACGACCAGAGGCATAC-3'. AdipoR1, forward, 5'-AACGGGCCATCCATTTTTG-3' and reverse, 5'-TTAGCCGGG CTACATCAAGG-3'. AdipoR2, forward, 5'-AGTGTTTTCAGCACGCCCTC-3' and reverse, 5'-GCTGAGCTCCACGGATTCTT-3'. The mRNA levels were obtained from the value of threshold cycle (*C*t) for each specific gene and normalized against the *C*t of β-actin.

### SiRNA Mediated silencing of TLR2 and TLR4

Adipocytes at day 6 into differentiation were transfected with the Deliver X^® ^transfection reagent (Panomics, Redwood City, CA) with 50 nM siRNA duplexes of either TLR4 (5'-CCCAAUUGA CUUCAUUCAAGATT-3' and 5'-UCUUGAAUGAAGUCAAUUGGGTT-3') or TLR2, (5'-CAAAGUGGUUGUCGCCUGCUUUTT-3' and 5'-AAGCAGGCGACAACC ACUUUGTT-3') for 72 hours. A non-silencing SiRNA duplex (vesirna) (Ambion, Austin, TX) was used as negative control. Cells were used for experiments immediately after the end of transfection (Day 9 post differentiation).

### Pretreatment of adipocytes with specific pharmacological inhibitors

To investigate the role of the specific signaling pathways in the regulation of peptidoglycan mediated cellular responses, cells were pretreated for 1 hour with 10 μM of U0126, SP600125 (abbreviated SP) and 50 μg/ml SN50 (Biomol, Plymouth Meeting, PA), specific inhibitors for p44/42 mitogen activated protein kinase (ERK/p44/42MAPK), c-Jun N terminal kinase (JNK) and nuclear factor kappa B (NFκB) respectively. Adipocytes were subsequently treated with LPS or peptidoglycan for the indicated periods.

### Fatty Acid Regulation of TLR2 and TLR4

Because obesity is associated with elevated levels of fatty acids, we investigated whether fatty acids directly regulate TLR2 and TLR4 mRNA expression. Adipocytes were treated with 500 μM linoleic acid (LIN) for 6 hours alongside LPS and peptidoglycan. Additional studies were conducted to determine the effect of Omega 3 fatty acids docosahexaenoic acid (DHA) and eicosapentaenoic acid (EPA) on TLR2 mRNA expression. Adipocytes were pretreated with 500 μM of each fatty acid for 3 hours before LPS or peptidoglycan treatment for another 6 hours. Fatty acids were complexed with BSA (3:1) before being used, and BSA was added to control treatments that did not receive fatty acids.

### ELISA for Media IL6

Media concentrations of IL6 were determined in duplicates per sample using a mouse IL6 ELISA kit (Endogen, Rockford, IL) according to the manufacturer's instructions. This kit has an assay sensitivity of < 7 pg/ml and an inter assay and intra assay variation of < 10%.

### Statistical Analyses

All data were checked for normality and then analyzed using the general linear model (GLM) of SAS. When there was a significant treatment effect as indicated by the F statistic, a mean separation analysis was performed with the least-squares mean separation procedure.

## Results

### Adipocytes respond directly to TLR2 activation by peptidoglycan and upregulate the expression and secretion of IL6

**Figure 1:**
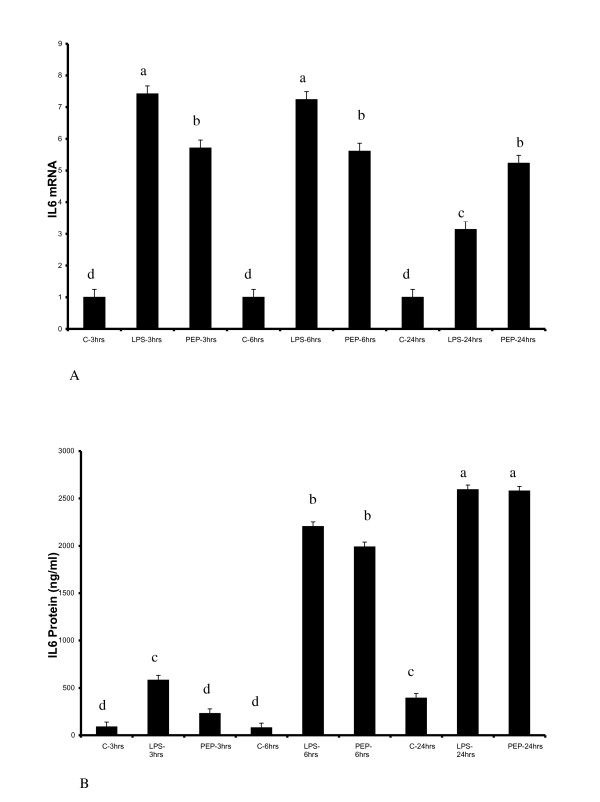
**Lipopolysaccharide (LPS) and peptidoglycan (PEP) induce IL6 mRNA expression and protein secretion**. Cells were treated with 100 ng/ml LPS and 10 μg/ml peptidoglycan (PEP) for 3, 6 and 24 hours. Media and mRNA were recovered for each time period and assayed for IL6 gene expression using RT-PCR (1A) and protein secretion with ELISA (1B). mRNA and media concentration of IL6 were increased by LPS and PEP. Bars represent means and ± SEM of this normalized expression from 4 different replicates. Bars with different superscripts are different (P < 0.05).

Since IL6 represents a major inflammatory cytokine that is over expressed in adipocytes in obesity, we first sought the possibility of its induction in response to peptidoglycan. As presented in Figure 1A and 1B, both peptidoglycan and LPS stimulate a significant upregulation of IL6 mRNA expression and secretion respectively (P < 0.05). Interestingly, whereas there was a fall in the level of IL6 mRNA in LPS treated cells after 24 hours of exposure, IL6 mRNA expression was sustained for the entire 24 hours in peptidoglycan treated cells. This may suggest fundamental differences in the signaling characteristics of these receptors, with TLR2 having a chronic effect, longer than TLR4.

### TLR4 activation with LPS, but not TLR2 activation with peptidoglycan, is inhibited by endotoxin neutralization with polymyxin B

**Figure 2:**
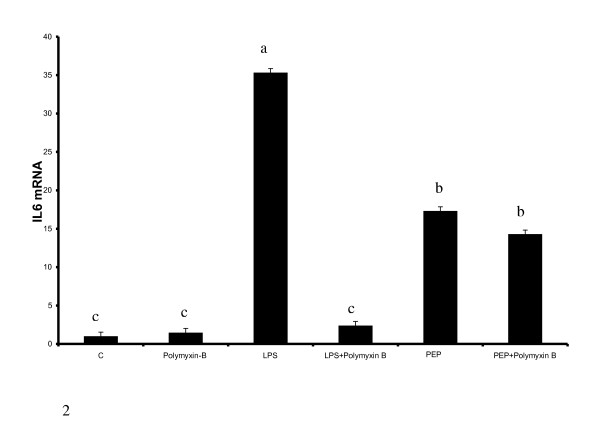
**Polymyxin B inhibits induction of IL6 mRNA by LPS, but had no effect against peptidoglycan**. Cells were treated with 100 μg/ml polymyxin B for 30 minutes before treatment with LPS (100 ng/ml) and PEP (10 μg/ml PEP) for 6 hours. Induction of IL6 gene expression by LPS treatment was significantly suppressed in the presence of polymyxin, but not PEP induction of IL6. Bars represent means and ± SEM of this normalized expression from 4 different replicates. Bars with different superscripts are different (P < 0.05).

To eliminate the possibility that endotoxin contamination could mediate the inflammatory response to peptidoglycan that was obtained, cells were pretreated with polymyxin B to neutralize LPS. As presented in Figure 2, sequestration of LPS with polymyxin completely abolishes the response to LPS, but does not inhibit peptidoglycan effect. This further supports our hypothesis that the response to peptidoglycan was not mediated by LPS contamination.

### Immunoneutralization of TLR2 with a neutralizing antibody and suppression of TLR2 with TLR2 specific siRNA abolishes inflammatory response to peptidoglycan

**Figure 3:**
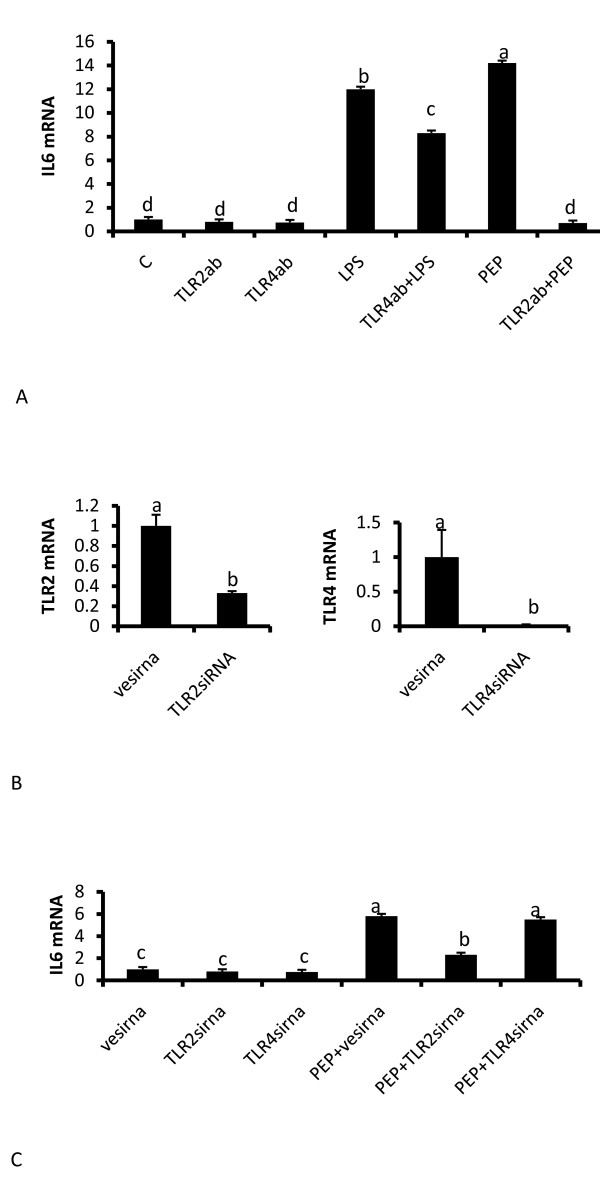
**Effect of immunoneutralization of TLR4 and TLR2 with their respective antibodies and siRNA mediated suppression of TLR2 and TLR4 on IL6 expression**. Cells were treated with 5 μg/ml neutralizing antibodies against TLR2 (TLR2ab) and TLR4 (TLR4ab) for 1 hour before treatment with LPS (100 ng/ml) and PEP (10 μg/ml) for 6 hours (3A). Both antibodies inhibit (P < 0.05) the induction of IL6 mRNA by their respective receptors. In cells treated with TLR2 and TLR4 specific siRNAs expression of TLR2 and TLR4 was reduced by their respective siRNAs (3B). Induction of IL6 expression by treatment with peptidoglycan was attenuated in cells treated with TLR2 siRNA, but not the negative siRNA (vesirna) or TLR4 siRNA (3C). Bars represent means and ± SEM of this normalized expression from 4 different replicates. Bars with different superscripts are different (P < 0.05).

Although TLR2 is the recognized receptor for peptidoglycan, adipocytes express multiple toll receptors and other classes of scavenger receptors. Therefore, to confirm that the inflammatory response to peptidoglycan was specific to TLR2, first we neutralized TLR2 with an immunoneutralizing antibody. As shown in Figure 3A, inhibiting TLR4 with its antibody inhibits the response to LPS as expected and neutralizing TLR2 completely suppresses the response of adipocytes to peptidoglycan (P < 0.05). In another set of experiments, suppression of TLR2 and TLR4 with their respective siRNAs (Figure 3B) leads to reduction in the mRNA of both receptors (P < 0.05). However, only TLR2 specific siRNA prevents the response to peptidoglycan whereas TLR4 siRNA had no effect (Figure 3C) (P < 0.05).

### Regulation of peptidoglycan-induced IL6 gene expression by p44/42 MAPK, c-JNK and NFκB

**Figure 4:**
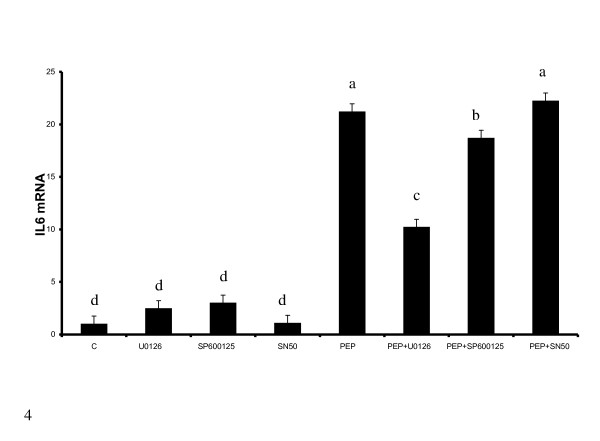
**Inhibition of extracellular signal regulated kinase (ERK) and JNK inhibits IL6 induction by peptidoglycan**. Both ERK and JNK were inhibited by treating cells with 10 μM of U0126 and SP600125(SP) and 50 μg/ml of the NFkB inhibitory peptide (SN50) for 30 minutes before treating with LPS (100 ng/ml) and PEP (10 μg/ml) for 6 hours. Inhibition of ERK and JNK, but not NFκB, inhibited IL6 induction by peptidoglycan. Bars represent means and ± SEM of this normalized expression from 4 different replicates. Bars with different superscripts are different (P < 0.05).

We also determined the effect of inhibiting extracellular signal regulated kinase (ERK), c-Jun N terminal Kinase (c-JNK) and the nuclear factor kappa B (NFκB) pathways on the induction of IL6 expression. Our previous work and that of others have shown that these pathways are important in the regulation of IL6 expression in response to TLR4 activation [1,15]. Inhibition of both the ERK and c-JNK pathways with their respective inhibitors (U0126 and SP600125 or SP) suppresses IL6 induction by peptidoglycan treated cells (Fig. 4A). However, inhibiting NFkB with the inhibitory peptide (SN-50) did not abrogate IL6 mRNA induction by peptidoglycan.

### Regulation of TLR2 and TLR4 mRNA expression

**Figure 5:**
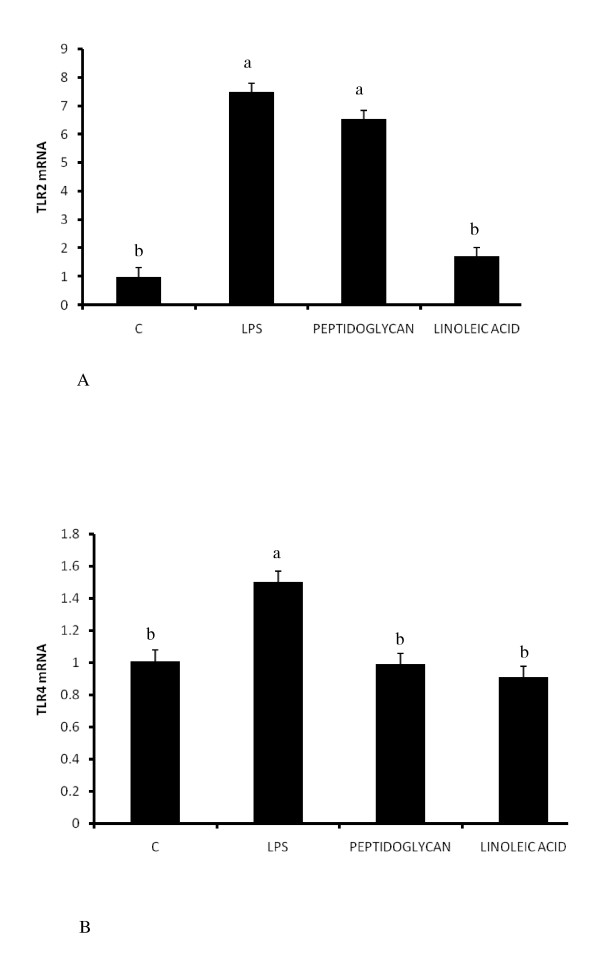
**Regulation of TLR2 and TLR4 mRNA expression by LPS, peptidoglycan and linoleic acid**. Expression of both TLR2 (5A) and TLR4 (5B) was determined in cells treated with LPS (100 ng/ml), peptidoglycan (10 μg/ml) and linoleic (500 μM) acid for 6 hours. Significant induction of TLR2 (5A) (approx.7 fold) and TLR4 (5B) (1.5 times) was obtained. Bars represent means and ± SEM of this normalized expression from 4 different replicates. Bars with different superscripts are different (P < 0.05).

**Figure 6:**
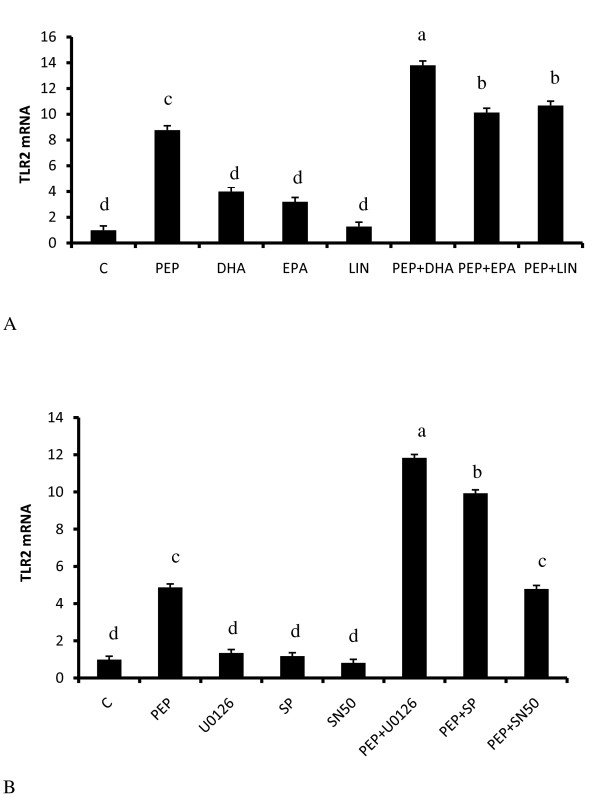
**Regulation of TLR2 induction peptidoglycan by fatty acids and pharmacological inhibitors of ERK (p44/42 MAPK), JNK and NFκB**. In cells pretreated with 500 μM linoleic acid (LIN), eicosapentaenoic acid (EPA) and docosahexaenoic acid (DHA) and then treated with peptidoglycan (6A), TLR2 mRNA expression was further additively upregulated in the presence of the fatty acids and peptidoglycan. Additionally, in cells were pretreated with the specific inhibitors, 10 μM U0126 and SP600125 (SP) and 50 μg/ml SN50 before peptidoglycan treatment for 6 hours. Inhibition of ERK and JNK, but not NFκB (6B), additively upregulated peptidoglycan induced TLR2 expression. Bars represent means and ± SEM of this normalized expression from 4 different replicates. Bars with different superscripts are different (P < 0.05).

We also examined the regulation of TLR2 and TLR4 mRNA expression in response to both LPS and peptidoglycan to determine if these were subject to regulation to in response to their respective ligands and fatty acids. Whereas TLR2 mRNA expression was induced 7 fold by both LPS and peptidoglycan (Fig. 5A) (P < 0.05), only minimal upregulation of TLR4 mRNA was obtained (1.5 fold) (Fig. 5B), and in LPS treated cells only. Linoleic acid treatment did not affect the expression of both receptors. Because TLR2 was robustly induced by both LPS and peptidoglycan, we examined whether its mRNA expression was subject to regulation by fatty acids. In adipocytes treated with DHA and EPA (Fig. 6A), small but significant (P < 0.05) induction of TLR2 mRNA was observed and both DHA and EPA treated cells. However, all three fatty acids additively led to increased TLR2 mRNA expression in conjunction with peptidoglycan (P < 0.05).

### Inhibition of p44/42 MAPK and c-JNK upregulates TLR2 mRNA

We determinedTLR2 mRNA expression in cells pretreated with specific inhibitors against p44/42 MAPK, c-JNK and NFκB. Unlike the case with IL6 expression which was inhibited when p44/42 and c-JNK were inhibited, there was an upregulation of TLR2 in the presence of peptidoglycan and inhibitors to both p44/42 MAPK and c-JNK (Fig. 6B). However, as observed for IL6 expression, inhibition of NFκB did not affect TLR2 expression.

### Peptidoglycan downregulates adiponectin receptors 1 and 2 expression

**Figure 7:**
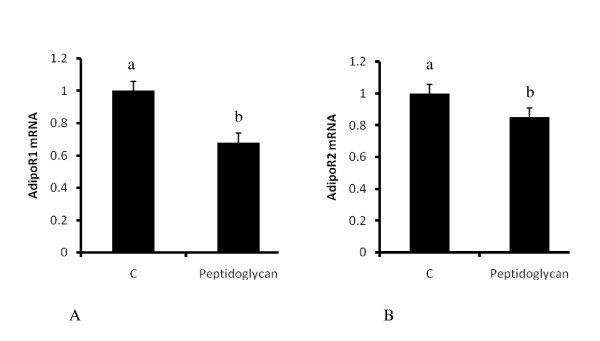
**Regulation of adiponectin receptors, adipoR1 and adipoR2, by peptidoglycan**. Acute treatment of adipocytes with peptidoglycan (10 μg/ml) for 3 hours leads to a significant downregulation of adipoR1 (7A) and adipoR2 (7B). Bars represent means and ± SEM of this normalized expression from 3 different replicates. Bars with different superscripts are different (P < 0.05).

Because adiponectin is a major adipokine that is involved in stimulation of glucose uptake and fatty acid oxidation, we also examined the regulation of the expression of its receptors, adipoR1 and adipoR2, by peptidoglycan. Both receptors were significantly downregulated (Figure 7A and 7B) by adipocyte exposure to peptidoglycan (P < 0.05).

## Discussion

Adipose tissue plays a significant role in the response to inflammatory stimuli. This role is conserved from drosophila to mammals, and because of their strategic location around organs, adipocytes are able to participate in the recognition and neutralization of multiple pathogens. Thus adipocytes facilitate a robust innate immune defense system [1,24,25]. The innate immune response mediated by adipocytes is primarily mediated by adipokines released in response to inflammatory stimuli [26,27]. Although the expression of TLR2 in adipocytes suggests a capability to respond to TLR2 ligands, [5,7,10], little is known regarding the activation of inflammatory response by peptidoglycan in adipocytes. We have provided evidence herein that adipocytes respond directly to TLR2 activation with the peptidoglycan component of gram positive bacteria. The ability of adipocytes to recognize gram positive bacteria component fills a critical gap regarding the capability of adipocytes to neutralize both classes of bacteria pathogens and demonstrates the versatility of immune reaction mediated by the adipocytes. The recent evidence that fatty acids act as endogenous ligands for TLR2 in hypertrophic adipose tissue leading to the activation of a subset of adipose tissue macrophages [28] supports a relevant role for TLR2 in vivo. Since fatty acid concentrations are elevated in obesity, fatty acids could be the major endogenous ligands for TLR2 in adipose tissue. Whereas TLR4 activation induces a rapid acute response, the inflammatory response to TLR2, demonstrated by the induction of IL6 in our case, is slower, but surprisingly prolonged. Therefore, although activation of both receptors elicits an inflammatory response, TLR2 induces a chronic inflammatory state. Although peptidoglycan recognition protein (PGRP) and NOD have been shown to mediate peptidoglycan response in some cell types [29], their role in mediating the response obtained in this experiment is limited because immunoneutralization with a TLR2 specific antibody totally ablates response to peptidoglycan, and a TLR2 specific siRNA drastically (approx. 80%) reduces IL6 induction by peptidoglycan. Obesity and type 2 diabetes are associated with a chronic low grade systemic and adipose tissue inflammation [30,31]. Interestingly, obesity and type 2 diabetes are also associated with increased expression of TLR2, and obesity induces the expression of a subset of adipocytes that over express both TLR2 and TNFα [8,10]. The reduction in the expression of adiponectin receptors in response to TLR2 activation with peptidoglycan also agrees with the finding that these receptors are downregulated in adipose tissue of obese and insulin resistant mice [22]. This raises the possibility that activation of TLR2 in obesity may contribute to a state of adiponectin resistance in obesity. Because adiponectin exerts anti-inflammatory effects [16], a reduction in the expression of its receptors could attenuate this critical role of adiponectin. Therefore, TLR2 may be an important player in the perpetuation of inflammation that characterizes obesity.

Our work and that of others have shown that multiple signaling pathways mediate LPS induction of IL6 [1,23,32]. These pathways include NFκB, c-JNK, ERK, inhibitory G protein and PKC mediated processes. Toll like receptors activate similar but distinct signaling pathways due to their ability to recruit different adapter proteins. TLR2 is able to recruit TIRAP/Mal, and this allows it to regulate the expression of a distinct set of inflammatory genes such as IL6, TNFα and IL12 [33-35]. Our work has shown that inhibition of NFκB failed to suppress induction of IL6 by peptidoglycan and also had no effect on TLR2 mRNA expression. It is unknown whether this observation is unique to the 3T3-L1 adipocytes or related to our experimental conditions, but parallels the inability of NFκB inhibition to suppress IL6 induction by LPS in myocytes [23]. However, additional studies will be needed to fully characterize the uniqueness of the signaling characteristics of TLR2 in adipocytes in comparison with other toll receptors.

The strong induction of TLR2 mRNA by both LPS and peptidoglycan that was observed in this study clearly supports a role for TLR2 as a strong marker of inflammation in adipocytes. This corroborates the induction of TLR2, but not TLR4, by LPS in mouse splenic macrophages [35]. Since obesity is an inflammatory condition that is also associated with elevated TLR2 expression in adipose tissue, adipocyte TLR2 may indeed mediate part of the inflammatory environment that characterizes obesity [8,10,30]. Therefore, targeting TLR2 may contribute to prevention of obesity-induced inflammation. Since obesity is also associated with elevated fatty acid levels, the induction of TLR2 expression by both DHA and EPA individually and additively with peptidoglycan and the additive induction of TLR2 by both linoleic acid and peptidoglycan suggests that fatty acids may be partly responsible for the upregulation of TLR2 in obesity. This also suggests that regulation of TLR2 mediated cellular responses may be fatty acid specific. Elevated fatty acid concentrations in obesity may amplify the inflammatory cascade that is induced by yet unidentified endogenous ligands for TLR2. Although omega-3 fatty acids, EPA and DHA at moderate levels are known to exert anti-inflammatory effects, elevated levels of these fatty acids in circulation has been demonstrated to cause increased inflammation characterized by increased macrophage infiltration into adipose tissue [36]. Therefore, the levels of these fatty acids as utilized in this experiment mimic more closely the hyperlipidemic condition that is characterized by elevated fatty acid concentrations. The additivity of effects of fatty acids and peptidoglycan on the induction of TLR expression suggests that under the hyperlipidemic conditions of obesity fatty acids and ligands of TLRs may co-operate to amplify the inflammatory state by further increasing the expression of TLRs. This may be a mechanism to prevent desensitization to the effects of TLR ligands in obesity.

Interestingly, whereas inhibition of p44/42 MAPK and c-JNK suppressed peptidoglycan induction of IL6, it amplifies the induction of TLR2 mRNA by peptidoglycan. This observation agrees with the upreguation of TLR2 mRNA by p44/42 MAPK inhibition with PD 98059 in RAW 264.7 macrophages [35]. Furthermore, it indicates that these kinase pathways, although they mediate positively the induction of IL6 by peptidoglycan, they may also be involved in a negative feedback mechanism to prevent an upregulation of TLR2 during inflammation, perhaps to prevent on overzealous inflammatory reaction.

The downregulation of expression of both adiponectin receptors by peptidoglycan parallels the reduction of soluble adiponectin receptor expression after the administration of LPS to human subjects [37] and suggests that TLR2 activation in obesity may partly be responsible for the downregulation of adiponectin receptor expression in adipose tissue in obesity [22]. Therefore, this may implicate TLR2 in phenomenon of obesity-induced adiponectin resistance. Because reduced expression of adiponectin receptors correlates with reduced AMPK activation, TLR2 activation may play an active role in the worsening insulin resistance in obesity.

In conclusion, we have provided evidence that adipocytes are able to respond to gram positive bacteria component in a TLR2 dependent manner and the response to TLR2 stimulation appears to perpetuate a chronic immune response. Since TLR2 expression is induced in obesity and type 2 diabetes, TLR2 may play a prominent role in the initiation of obesity induced inflammation. The use of TLR2 knockout animal models will provide further insight into this mechanism. An understanding of the role of TLR2 and its interactions with other receptors in the adipocyte may yield significant insights into the regulation of inflammation in obesity and may help in the search for new therapeutic targets against obesity-induced impairment of insulin signaling.

## Abbreviations

TLR: Toll-like receptors; IL6: interleukin 6; LPS: lipopolysaccharide; PEP: Peptidoglycan; MAPK: Mitogen activated protein kinase; c-JNK: c-Jun N terminal kinase; adipoR1 and adipoR2: adiponectin receptor1 and 2; NfκB: Nuclear factor kappa B; ELISA: Enzyme linked immunoabsorbent assay.

## Competing interests

The authors declare that they have no competing interests.

## Authors' contributions

KA conceived the original research idea and executed the study. WB and TW assisted in getting the funds used and were involved in the writing and review of the manuscript.
